# Scientific Publications on the Association Between Obstructive Sleep Apnea Hypopnea Syndrome (OSAHS) and Metabolic Dysfunction-Associated Steatotic Liver Disease (MASLD): A Bibliometric Study From 2010 to 2024

**DOI:** 10.7759/cureus.90708

**Published:** 2025-08-21

**Authors:** Jeysson E Mejia-Guzman, Francisco Fernández-Nogueira, Guadalupe Yarza-Rosas, David E González-Mendoza, José M Juárez-Sosa, Laureano Palacio-Zurita

**Affiliations:** 1 Health Sciences Academic Division, Universidad Juárez Autónoma de Tabasco, Villahermosa, MEX; 2 Traslational Research Unit, Medica Sur Clinic & Foundation, Mexico City, MEX; 3 Faculty of Medicine, Universidad Popular Autónoma del Estado de Puebla, Puebla, MEX; 4 Department of Otorhinolaryngology, Instituto Nacional de Rehabilitación Luis Guillermo Ibarra Ibarra, Mexico City, MEX; 5 Mexican Faculty of Medicine, Universidad La Salle, Mexico City, MEX; 6 Faculty of Medicine, Autonomous University of Mexico State, Toluca, MEX; 7 Faculty of Medicine, Meritorious Autonomous University of Puebla, Puebla, MEX; 8 Department of Otorhinolaryngology, Medica Sur Hospital, Mexico City, MEX

**Keywords:** bibliometric analysis, hepatic steatosis (masld), nonalcoholic fatty liver disease (nafld), obstructive sleep apnea hypopnea syndrome, scientific publication indicators, visual analysis

## Abstract

This bibliometric review analyzed global research trends on the relationship between obstructive sleep apnea hypopnea syndrome (OSAHS) and metabolic dysfunction-associated steatotic liver disease (MASLD) from 2010 to 2024. A total of 115 PubMed-indexed publications were identified using Medical Subject Headings (MeSH)-based search strategies, with citation counts and journal impact factors obtained from Web of Science. The analysis considered multiple bibliometric indicators, including publication counts, citation frequencies, author productivity, journal impact, country contributions, and publication languages. Over the study period, research output increased at a compound annual growth rate (CAGR) of 5.1%, peaking in 2021 with 13 publications. The average number of citations per year was 421.3, with a peak in 2013 (910 citations) and a citation CAGR of −23.9%, a pattern likely influenced by the shorter citation window of recent studies. Research activity was dominated by China and the United States, while no publications originated from Spanish-speaking countries despite the high regional prevalence of both conditions. These findings reveal a pronounced geographical imbalance in the literature, emphasizing the urgent need to stimulate research in underrepresented regions. Furthermore, the recent nomenclature transition from non-alcoholic fatty liver disease (NAFLD) to MASLD is expected to influence future bibliometric trends, requiring hybrid search strategies to ensure continuity in literature tracking during the transitional period.

## Introduction and background

Despite the high clinical and epidemiological burden of obstructive sleep apnea hypopnea syndrome (OSAHS) [1] and metabolic dysfunction-associated steatotic liver disease (MASLD) [2,3], no bibliometric analysis has mapped the global scientific production on their interrelationship, creating a clear knowledge gap that this study seeks to address.

OSAHS is a common chronic respiratory disorder characterized by episodes of complete obstruction (apnea) or partial obstruction (hypopneas) of the upper airways intermittently and repeatedly during sleep. These episodes occur more than five times per hour and are mostly associated with hemoglobin oxygen desaturations accompanied by sympathetic hyperactivity, which eventually cause sleep disruption and consequently fatigue and daytime somnolence [4,5]. It is estimated that approximately one billion adults aged 30-69 years worldwide suffer from moderate to severe OSAHS. This disorder affects 34% of men and 17% of women in the general population [6].

In June 2023, an international agreement was reached that proposed modifying the nomenclature of non-alcoholic fatty liver disease (NAFLD) to MASLD [7-9]. This change aims to emphasize the connection with cardiometabolic risk factors and, likewise, eradicate the stigma implied by the term "non-alcoholic". MASLD is operationally defined as the presence of hepatic steatosis accompanied by a cardiometabolic risk factor, which includes obesity, type 2 diabetes, dyslipidemia, or hypertension, or treatment for any of these. An extra criterion is established that alcohol consumption should be less than 140 grams per week for women and 210 grams for men [10,11].

**Figure 1:**
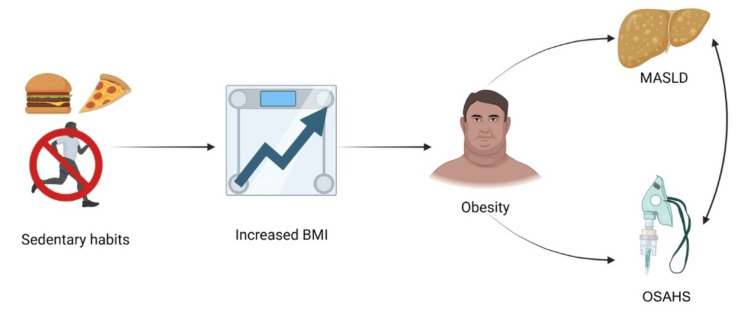
The relationship between MASLD and OSAHS Increased body mass index, resulting from sedentary lifestyles, contributes to obesity, consequently leading to MASLD and OSAHS, conditions that demonstrate interdependence. OSAHS: obstructive sleep apnea hypopnea syndrome; MASLD: metabolic dysfunction-associated steatotic liver disease Image Credit: Authors; created with BioRender.com

Obesity is a major risk factor for developing MASLD. Some of the pathophysiological mechanisms include insulin resistance, adipose tissue dysfunction, and tissue hypoxia. Hypoxia in adipose tissue is induced by obesity, which causes chronic inflammation and metabolic dysfunction that contribute to the progression of MASLD [1]. Furthermore, obesity is associated with an increased release of fatty acids from adipose tissue to the liver, which increases fatty acid production and reduces their oxidation in the liver, favoring hepatic fat storage (Figure 1) [12].

Bibliometrics is defined as a type of analysis that integrates qualitative and quantitative approaches, designed to examine academic literature. This analysis allows describing the temporal evolution of a specific topic, identifying trends in publications, and evaluating the productivity of both authors and countries. Additionally, in certain cases, it facilitates the prediction of future patterns in the literature [13].

MeSH (Medical Subject Headings) is a thesaurus developed by the United States National Library of Medicine; it is characterized by being expandable, dynamic, and standardized. This resource contributes to providing uniformity and consistency in the indexing and categorization of biomedical literature [14]. Given the emerging link between OSAHS and MASLD and the lack of bibliometric studies on the topic, this study aims to fill that gap by analyzing global publication trends from 2010 to 2024.

## Review

Materials and methods

Search Strategy

In the present study, we focused exclusively on publications related to OSAHS and MASLD available in the PubMed database, which houses numerous research journals and provides access to a wide range of publications. A notable feature of this database is its integration with tools like MeSH. An initial search was conducted to determine whether bibliometric studies combining OSAHS and MASLD had been previously published, testing combinations of MeSH terms such as (“Bibliometrics”\[Mesh]) AND “Non-alcoholic Fatty Liver Disease”\[Mesh], (“Sleep Apnea, Obstructive”\[Mesh]) AND “Bibliometrics”\[Mesh], and (“Bibliometrics”\[Mesh]) AND “Sleep Apnea Syndromes”\[Mesh]. No bibliometric study addressing both topics simultaneously was identified.

Given this gap, it was considered pertinent to conduct a bibliometric analysis of articles addressing the relationship between OSAHS and MASLD using the MeSH strategy ((“Sleep Apnea, Obstructive”\[Mesh]) AND “Sleep Apnea Syndromes”\[Mesh]) AND “Non-alcoholic Fatty Liver Disease”\[Mesh]. The search was performed on September 28, 2024, at 1:30 a.m. (Mexico City time), and retrieved 130 publications dated from January 1, 2010, to September 28, 2024.

Inclusion and Exclusion Criteria

For inclusion, only peer-reviewed original research articles, narrative and systematic reviews, and meta-analyses were considered. Editorials, letters, case reports, conference abstracts, and non-peer-reviewed materials were excluded. Although publications in any language were eligible, only English, Chinese, and French articles met the selection criteria.

Screening and Selection Process

**Figure 2:**
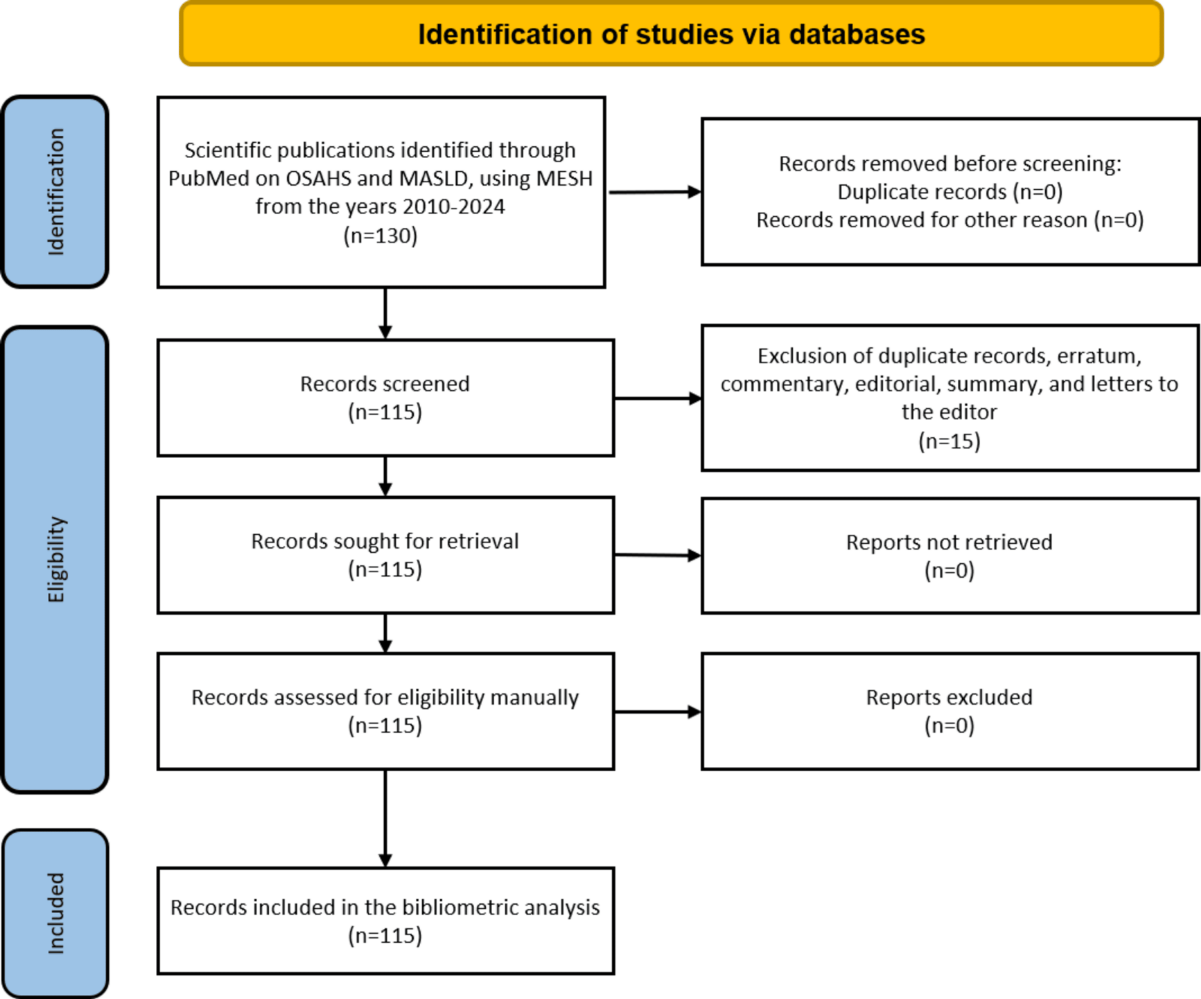
PRISMA-like flowchart of the study selection process. OSAHS: obstructive sleep apnea hypopnea syndrome; MASLD: metabolic dysfunction-associated liver disease; MESH: medical subject headings; PRISMA: Preferred Reporting Items for Systematic Reviews and Meta-Analyses

No duplicate records were found. Titles and abstracts were independently screened by two reviewers (JEMG and DEGM) to assess eligibility, and discrepancies were resolved by consensus. After applying the inclusion and exclusion criteria, 15 records were excluded for being conference abstracts, case reports, or editorials, resulting in a final set of 115 articles included in the analysis. The overall selection process is summarized in Figure 2 using a PRISMA-like flowchart format.

Data Extraction and Bibliometric Indicators

For each included article, information was extracted on the title, PMID, journal name, 2023 journal impact factor, number of citations, article type, country of origin, language, and year of publication. Citation counts and impact factors were obtained from the Web of Science database, and data were managed and analyzed in Microsoft Excel (Microsoft Corporation, Redmond, Washington, United States).

Database Rationale

PubMed was selected as the primary search source due to its standardized MeSH indexing and focus on high-quality biomedical literature. While other databases such as Scopus and Embase offer broader coverage, PubMed provides precise retrieval with consistent taxonomy, which is essential for bibliometric accuracy. Web of Science was chosen for its robust and standardized citation metrics.

Results

From 2010 to 2024, a total of 115 publications related to the association between OSAHS and MASLD were identified. The highest research output was observed between 2020 and 2022, followed by a decline in the number of publications in subsequent years, approaching the levels seen at the beginning of the study period when the first paper was published in 2011. The annual growth rate in scientific production, calculated as the compound annual growth rate (CAGR), was 14.1% over the entire study period.

**Figure 3:**
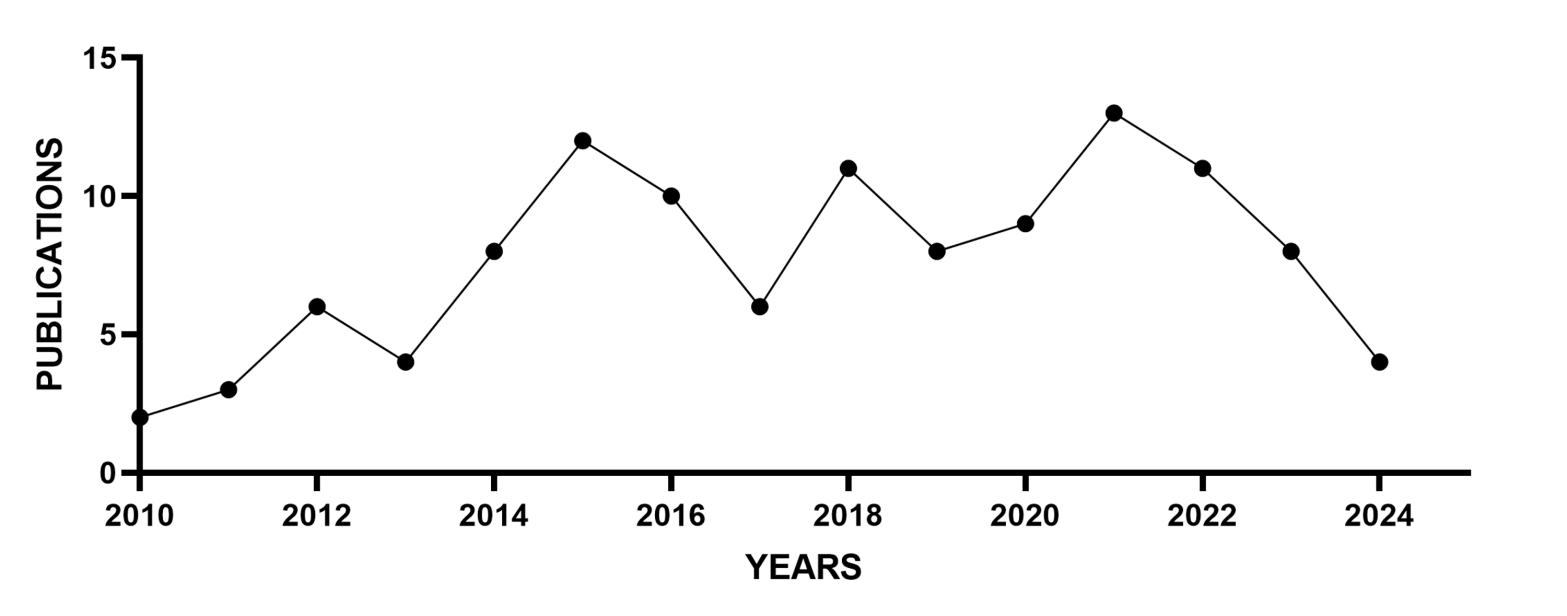
Scientific production by year from 2010 to 2024 Trends in publication rates on OSAHS and MASLD over time (2010-2024), developed based on information compiled and analyzed in the present bibliometric study. OSAHS: obstructive sleep apnea hypopnea syndrome; MASLD: metabolic dysfunction-associated liver disease

Figure 3 shows the annual distribution of publications, where a progressive increase can be observed until reaching the peak period, followed by a recent decrease.

Regarding citation dynamics, the 115 articles accumulated a total of 5,019 citations, with an average of 43.6 citations per publication. The year 2013 registered the highest citation count (910 citations), followed by 2012 (657 citations) and 2015 (657 citations). The compound annual growth rate in citations during the study period was 16.8%, reflecting increasing academic interest until recent years.

**Figure 4:**
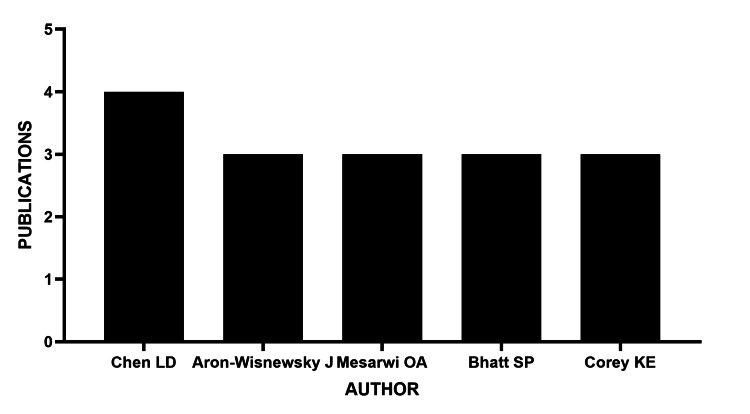
Authors with the highest number of publications on OSAHS and MASLD OSAHS: obstructive sleep apnea hypopnea syndrome; MASLD: metabolic dysfunction-associated steatotic liver disease

The most prolific author was Chen LD, with the highest number of publications on the topic, followed by Aron Wisnewsky, Mersawi OA, Bhatt SP, and Corey KE, each with three publications (Figure 4).

**Figure 5:**
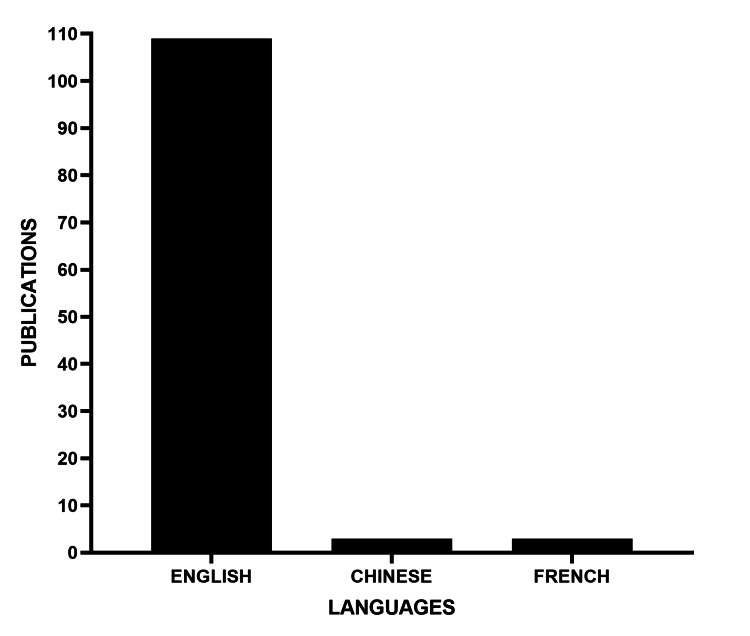
Number of publications by language

In terms of language, the vast majority of publications were in English (over 100), followed by Chinese and French. Notably, although the topic has been minimally studied in Mexico, no publication in Spanish was found during the analyzed period (Figure 5).

**Figure 6:**
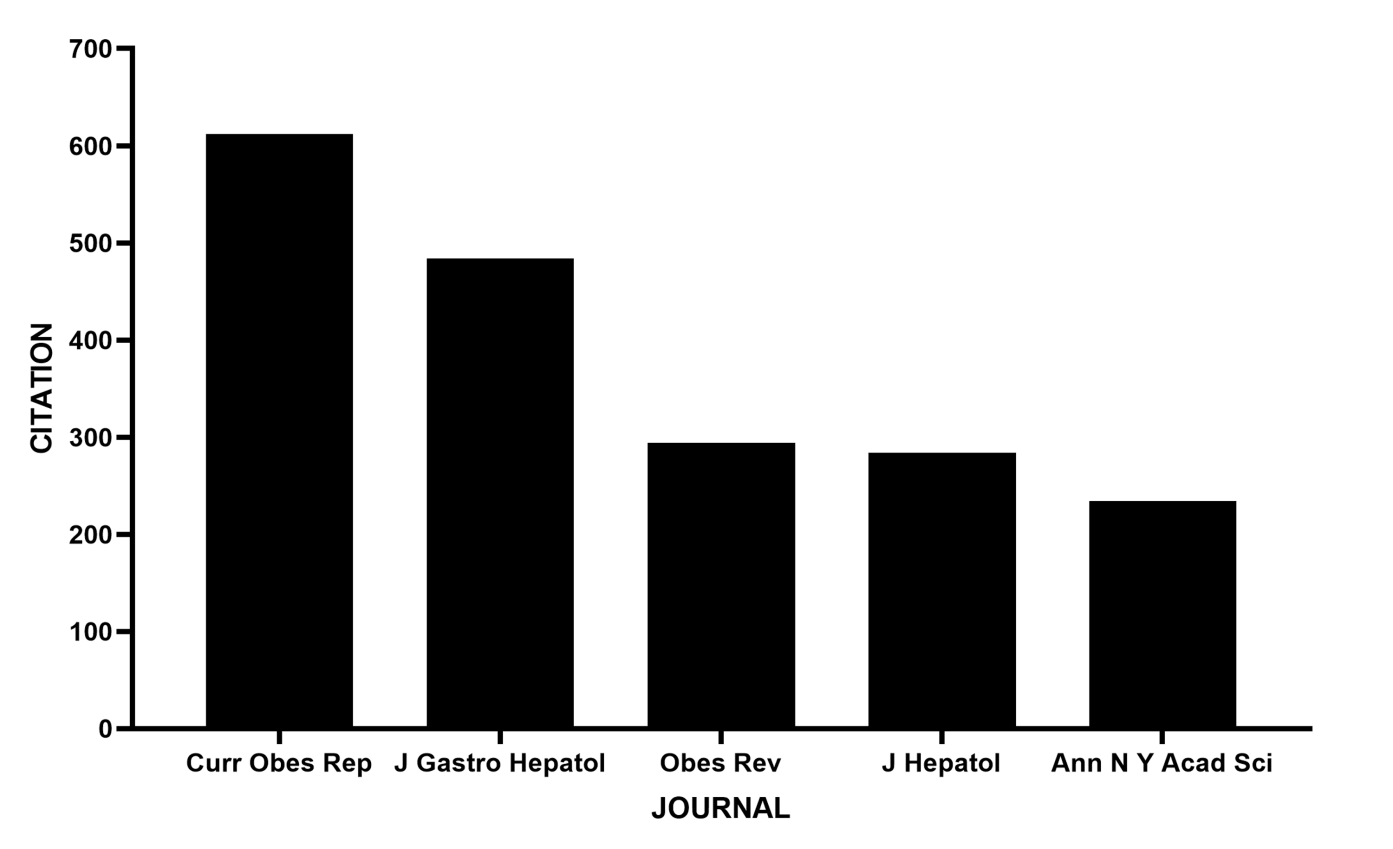
Journals with the highest number of citations to articles on MASLD and OSAHS OSAHS: obstructive sleep apnea hypopnea syndrome; MASLD: metabolic dysfunction-associated steatotic liver disease

The most cited publication is one that was published in the journal *Current Obesity Reports* with more than 600 citations to date, followed by the *Journal of Gastroenterology and Hepatology* with approximately 500 citations (Figure 6).

**Figure 7:**
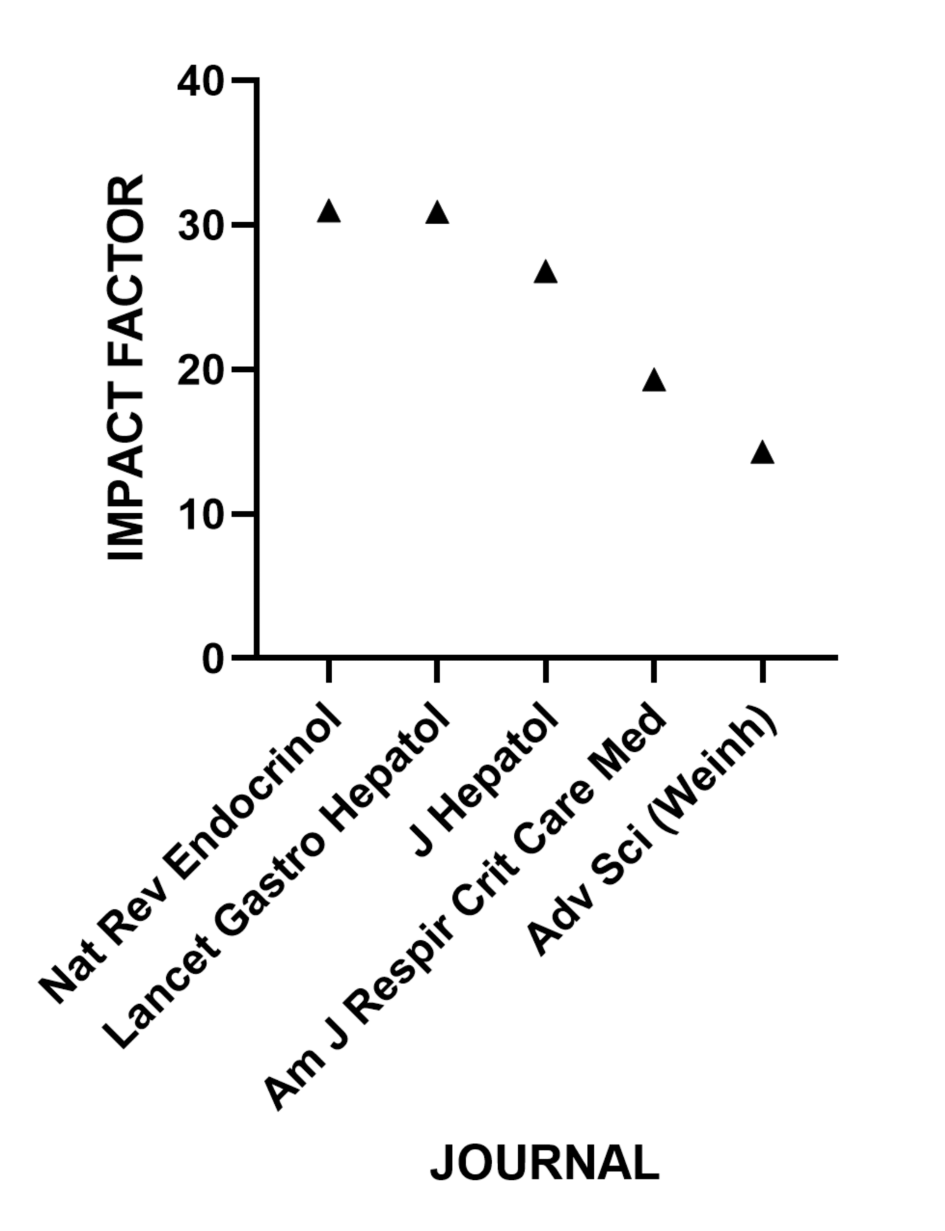
Journals publishing OSAHS and MASLD articles with the highest impact factors OSAHS: obstructive sleep apnea hypopnea syndrome; MASLD: metabolic dysfunction-associated steatotic liver disease

Among the journals covering the topic, the highest impact factor in 2023 was held by *Nature Reviews Endocrinology* and The *Lancet Gastroenterology* *and* *Hepatology* (impact factor: 30) (Figure 7).

**Figure 8:**
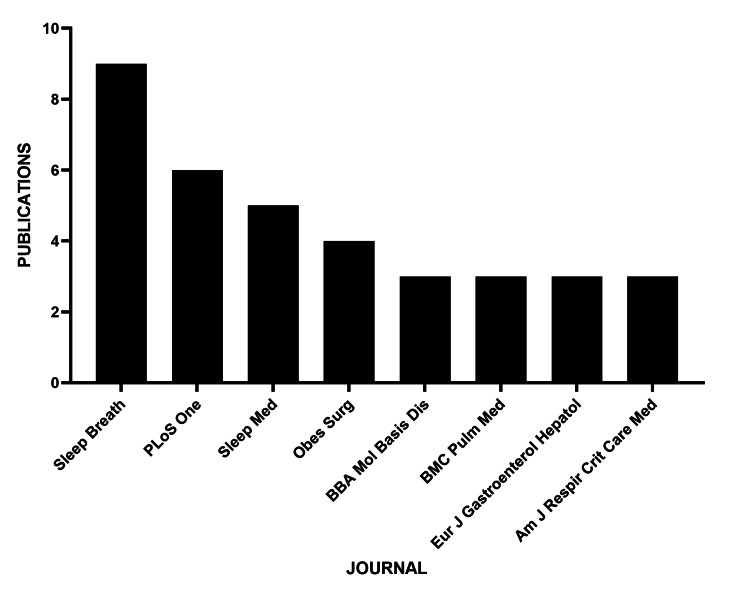
Journals with the largest number of publications on MASLD and OSAHS OSAHS: obstructive sleep apnea hypopnea syndrome; MASLD: metabolic dysfunction-associated steatotic liver disease

The journal with the largest number of publications on the subject was *Sleep and Breathing* (n = 9), followed by *PLOS ONE* (n = 6) (Figure 8).

**Figure 9:**
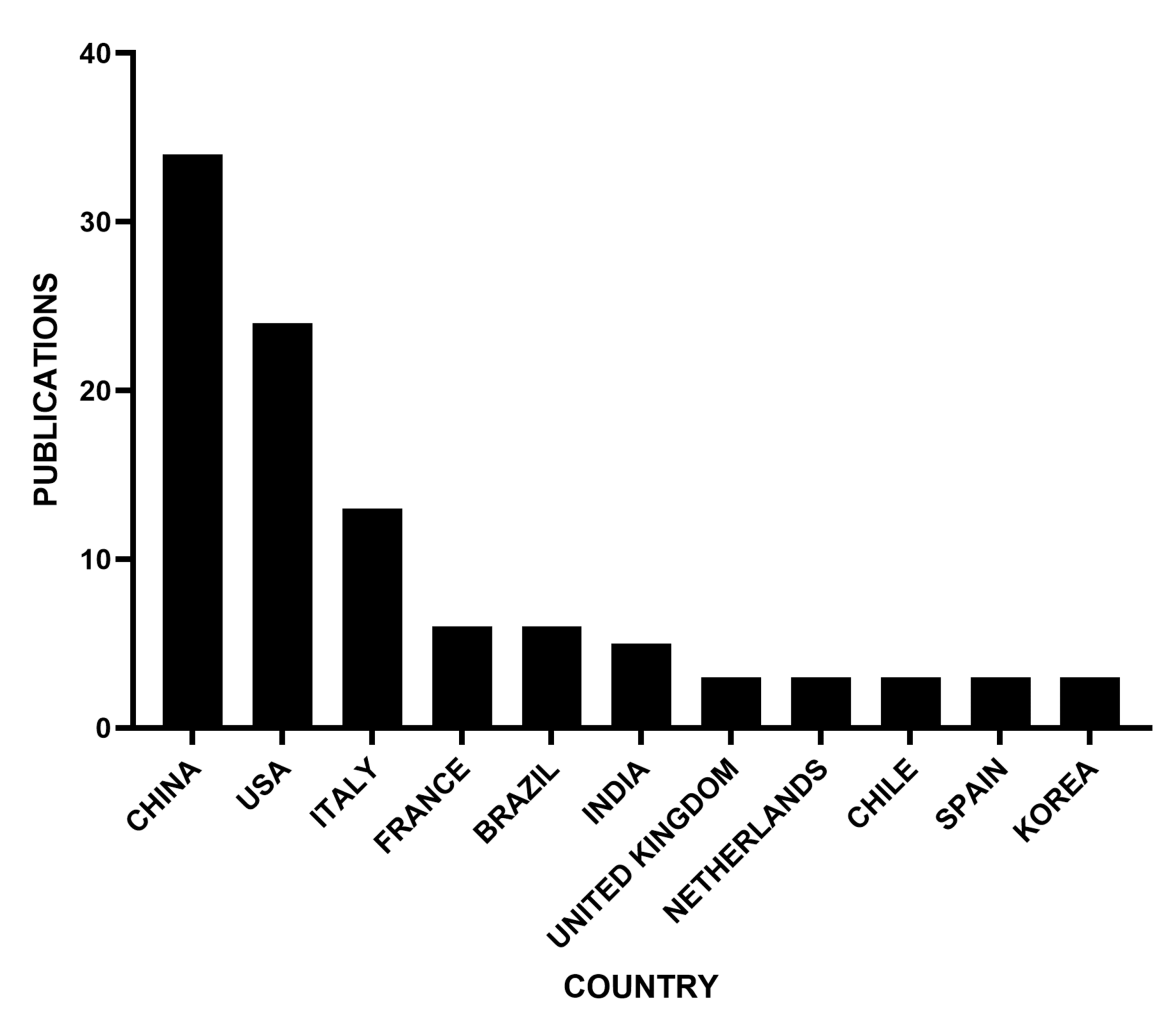
Countries with the highest production of MASLD and OSAHS publications OSAHS: obstructive sleep apnea hypopnea syndrome; MASLD: metabolic dysfunction-associated steatotic liver disease

The country that published the most research on the topic internationally is China, with more than 30 publications, followed by the United States (Figure 9).

**Figure 10:**
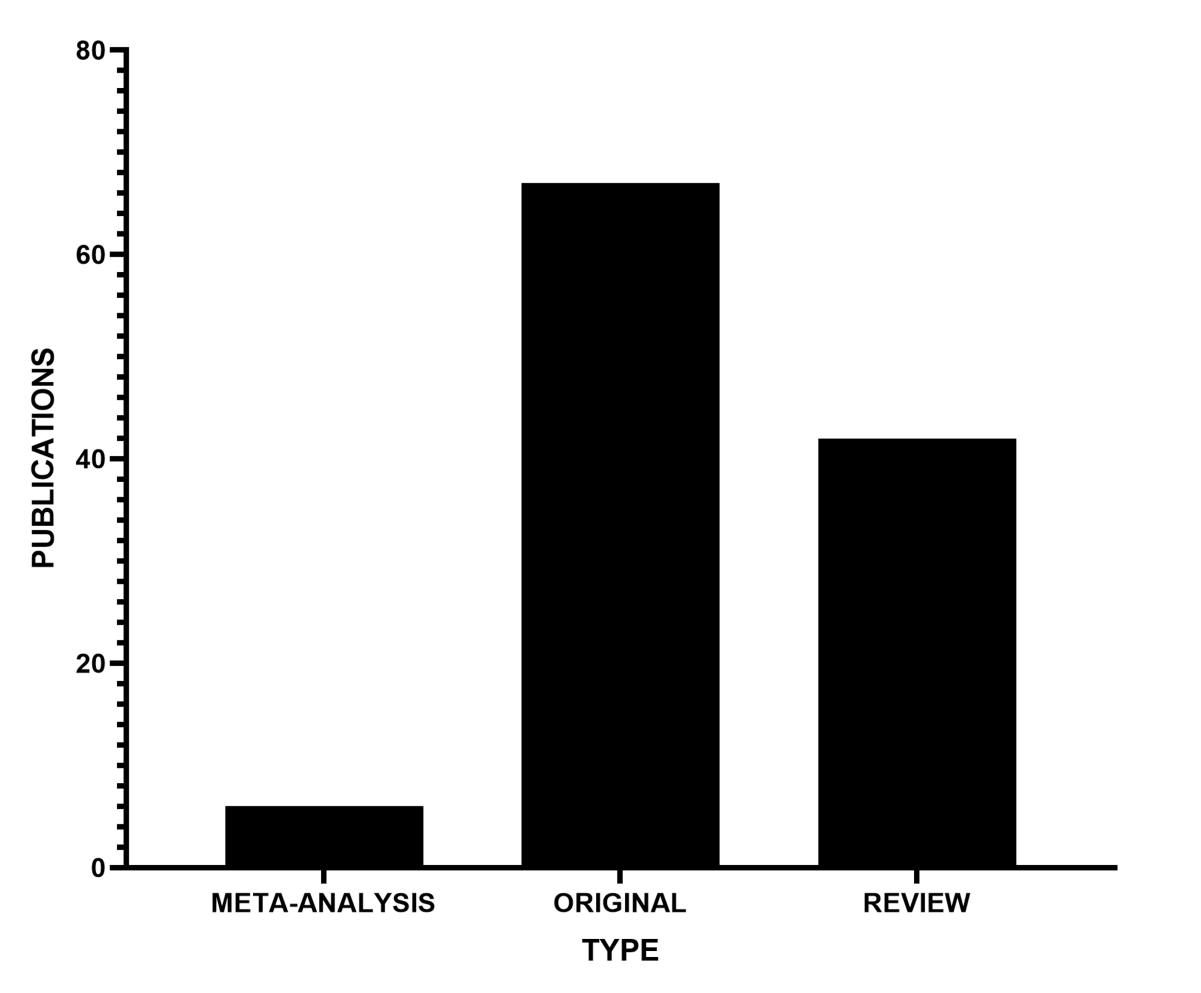
Number of publications by type

Lastly, original article was the most common type of publication, accounting for 70 of the total publications found, followed by review articles (Figure 10).

Discussion

This bibliometric study offers an overview of scientific research exploring the relationship between OSAHS and MASLD during the period 2010-2024. The analysis of 115 identified publications reveals significant patterns in academic production, highlighting a marked increase in research interest between 2020 and 2022, followed by a stabilization that likely reflects both the consolidation of knowledge and methodological challenges associated with the recent nomenclature change from NAFLD to MASLD in 2023 [5]. This terminological shift seeks not only to eliminate the stigma associated with the term "non-alcoholic" but also to more clearly emphasize the metabolic components of the disease, which could explain the slight decrease in publications observed later, as researchers adapt their approaches to the new diagnostic criteria.

The study's findings underscore the leadership of author Chen LD in this field, who has not only been one of the most prolific but has also contributed significantly to clarifying the underlying pathophysiological mechanisms between both conditions. His focus has particularly centered on the role of intermittent hypoxia, characteristic of OSAHS, in the exacerbation of hepatic steatosis through the induction of oxidative stress, mitochondrial dysfunction, and systemic inflammatory responses [9]. This reinforces the idea that the interconnected mechanisms between OSAHS and MASLD represent a shared pathogenic pathway of great clinical relevance.

The journals *Current Obesity Reviews *and *Nature Reviews Endocrinology* emerge as the main vehicles for disseminating this knowledge, reflecting the interdisciplinary nature of the research, which connects the fields of sleep medicine, hepatology, and endocrinology [8]. However, the largest number of publications were identified in journals such as *Sleep Breath* and *Plos One*, which have a lower impact factor. This discrepancy between volume and scientific visibility deserves attention in future dissemination strategies.

Geographically, China and the United States dominate the research landscape, representing over 50% of the analyzed publications, which contrasts sharply with the scarce representation of Latin American countries and other Spanish-speaking regions. This low scientific production is paradoxical, considering the high prevalence of both conditions in these populations, and may be explained by persistent barriers in access to funding, research infrastructure, and publication in high-impact journals indexed in major databases [2,3]. Strengthening regional research networks and promoting bilingual or multilingual publication policies could help narrow this gap and diversify the global evidence base.

From a pathophysiological perspective, the findings support the existence of interrelated mechanisms that closely link OSAHS with MASLD. Intermittent hypoxia is a central phenomenon in the pathophysiology of OSAHS that induces the mobilization of free fatty acids from adipose tissue, contributing to lipid overload in hepatocytes. In turn, it generates endoplasmic reticulum stress and activates the inflammatory response, facilitating the progression from simple steatosis to more advanced forms of liver damage. Available evidence also points to sustained activation of the sympathetic nervous system and the elevation of proinflammatory cytokines such as TNF-α and IL-6, which perpetuate metabolic dysfunction and liver injury [7,12].

Among the limitations of the present study is its restriction to the PubMed database for article retrieval. This methodological decision, while ensuring homogeneity, may have excluded relevant contributions indexed in other databases. Although our search strategy did not impose language restrictions, the resulting body of literature from PubMed was predominantly in English, with only a few articles in Chinese and French. This limitation becomes particularly relevant in the Latin American context, where genetic, cultural, and socioeconomic factors can differentially influence the expression and progression of these diseases.

Looking ahead, the need to promote research in diverse and underrepresented populations is evident, as is the creation of international consortia that integrate standardized methodologies and allow for prospective longitudinal studies. These strategies could help clarify the temporal relationship between OSAHS and MASLD, as well as evaluate the therapeutic impact of interventions such as continuous positive airway pressure on the progression of liver disease [15].

This bibliometric study not only maps the current research landscape on OSAHS and MASLD but also identifies critical gaps and opportunities for advancement. The observed geographical disparity suggests the need to strengthen research capabilities in currently underrepresented regions, while the solid pathophysiological foundations established justify the implementation of integrated clinical approaches. As the medical community fully adopts the new MASLD nomenclature, a resurgence of research incorporating these updated criteria is predictable, which could open new avenues for understanding and managing these interrelated conditions that represent significant challenges for global public health.

## Conclusions

Although research on the association between OSAHS and MASLD is still limited, the observed growth in publications underscores the growing recognition of their clinical and public health relevance. The striking geographic imbalance, with scarce contributions from Spanish-speaking countries despite their high disease burden, highlights the urgent need to foster research in underrepresented regions. Looking ahead, the adoption of the MASLD nomenclature is expected to reshape future bibliometric trends and stimulate new lines of investigation that integrate metabolic, genetic, and regional perspectives. Consolidating these efforts will be crucial to strengthen global knowledge and promote more effective, inclusive clinical strategies.
